# Supplementary material for: The Second Derivative of Fullerene C60 (SD-C60) and Biomolecular Machinery of Hydrogen Bonds: Water-Based Nanomedicine
Source: Micromachines (Basel). 2023 Nov 25;14(12):2152. doi: 10.3390/mi14122152 (PMC10745578; doi:10.3390/mi14122152)
Supplement: Supplementary file 1 [file micromachines-14-02152-s001.zip › micromachines-2718797-supplementary.pdf]

Supplementary Materials:

THE SECOND DERIVATIVE OF FULLERENE C_{60} (SD-C_{60}) AND BIOMOLECULAR MACHINERY OF HYDROGEN BONDS: WATER-BASED NANOMEDICINE

Lidija R. Matija [1,*], Ivana Mladen Stankovic [1], Milica Puric [1], Milica Miličić [1,2], Danijela Maksimović-Ivanić [3], Sanja Mijatovic [3], Tamara Krajnović [3], Vuk Gordic [3] and Djuro Lj. Koruga [1,4,*]

1 Nano Lab, Department of Biomedical Engineering, Faculty of Mechanical Engineering, University of Belgrade, 11220 Belgrade, Serbia; imileusnic@mas.bg.ac.rs (I.M.S.); mpuric@mas.bg.ac.rs (M.P.); milica.milicic@tftnanocenter.rs (M.M.);
2 TFT Nano Center, 11050 Belgrade, Serbia
3 Institute for Biological Research Siniša Stanković—National Institute of the Republic of Serbia, University of Belgrade, 11000 Belgrade, Serbia; nelamax@ibiss.bg.ac.rs (D.M.-I.); sanjamama@ibiss.bg.ac.rs (S.M.); tamara.krajnovic@ibiss.bg.ac.rs (T.K.); vukgordic6@gmail.com (V.G.);
4 NanoWorld, Biomedical Photonic Lab, 11043 Belgrade, Serbia
* Correspondence: matijalidija@gmail.com or lmatija@mas.bg.ac.rs (L.R.M.); djuro.koruga@gmail.com or dkoruga@mas.bg.ac.rs (D.Lj.K.); Tel.: +381-62295236 (L.R.M.); +381-63287353 (D.Lj.K.)

Supplement (S1) :Hydrogen bonds

1. Let us observe the interaction between two isolated water molecules via non-covalent hydrogen bonds as displayed in Figure 1. The length of covalent hydrogen bond is 0.0984±0.0025 nm, and this will be declared in our model as unity (“1”), a point of reference with respect to the noncovalent hydrogen bond. In other words, we shall observe a change of the non-covalent hydrogen bond “x” (as the larger part of the system “x+1”) with respect to “1” (as the smaller part in the system “x+1”). Since the water molecules spontaneously organize and deconstruct themselves, i.e., they have the property of self-organization (harmonization), based on a stable covalent hydrogen bond “1“, the ratio “x” to “x+1” should be as “1” to “x” (the larger part with respect to the whole is related as the smaller part to the larger part). Based on the regular ratio of parts to the whole and the ratio between parts, the proportion is obtained x: (x+1) = 1: x, giving $x^2 = x + 1$, namely $x^2 - x - 1 = 0$. Solutions of this quadratic equation are $x_1 = 1.61803$, which is Φ, and $x_2 = -0.61803$, which is -φ. Therefore, we observe that, for two molecules to organize themselves spontaneously into a dimer, the non-covalent hydrogen bond should be 1.61803±0.025 greater than the covalent hydrogen bond. Thus, the oscillation process occurs within limits 1.59303 and 1.64303 of the value of the covalent hydrogen bond. This is in agreement with neutron experimental data [(Jeffrey, G.A. and Saenger,W., Hydrogen Bonding in biological Structures, Springer-Verlag, Berlin, 1991 and Heyrovska, R., Dependence of the length of the hydrogen bond on the covalent and cationic radii of hydrogen, and additivity of bonding distances. Chemical Physics Letters, 432: pp. 348-351, 2006)].

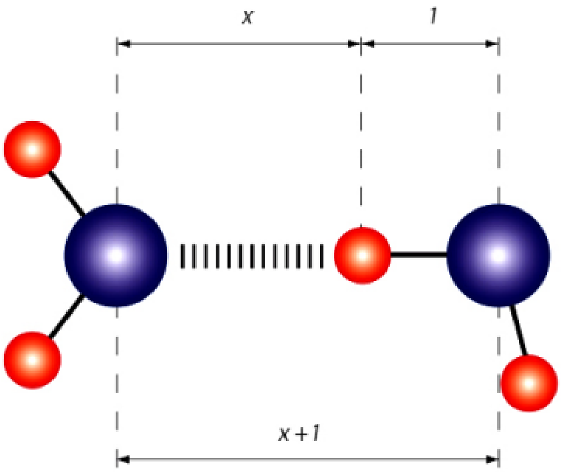

Supplement Figure S1**:** The interaction between two isolated water molecules via non-covalent hydrogen bonds, where "1" denotes the value of the covalent hydrogen bond (reference size), "*x*" is the variable distance between hydrogen atoms of one molecule and the oxygen atom of the other water molecule. The expression "x+1" represents the distance between centers of oxygen of two water molecule.

However, when we observe the organization of water molecules into a chain, as shown in Figure 2, then the notation for a water molecule is 1, for two molecules 1+1/1, for three molecules 1+ 1/(1+1), etc. We observe the system is getting more complex, and the displayed simplified organization of water molecules is only a possible variant. When we calculate the fractions from Figure 5.29, we obtain values 1, 2, 3/2, 5/3, 8/5 and 13/8. Thus, if we observe the water dimer, in case when two water molecules are at 13/8 Hyperpolarized Light the double value of the covalent hydrogen bond, they engage in a hydrogen noncovalent bond. It is known that the convergence point of this sequence is Φ (1.61803….)

A diagram representing values of water molecules organization into chains is displayed in Suppl. Figure 2, indicating that the establishing noncovalent hydrogen bond is the longest in a water dimer, equal to two lengths of the covalent hydrogen bond. When the bond is established and stabilized, the non-covalent hydrogen bond oscillates between values 1.615 and 1.625 and converged to Φ =1.61803….

$$1$$

$$1+\frac{1}{1}=2$$

$$1+\frac{1}{1+1}=\frac{3}{2}$$

$$1+\frac{1}{1+\frac{1}{1+1}}=\frac{5}{3}$$

$$1+\frac{1}{1+\frac{1}{1+\frac{1}{1+1}}}=\frac{8}{5}$$

$$1+\frac{1}{1+\frac{1}{1+\frac{1}{1+\frac{1}{1+1}}}}=\frac{13}{8}$$

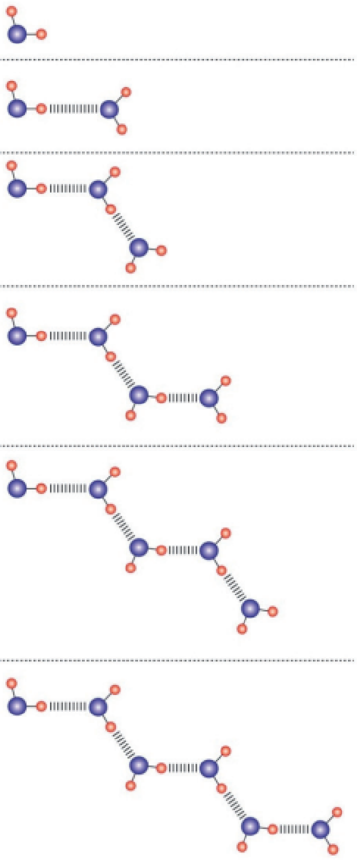

Supplement Figure 2 (S2): The Fibonacci fractions values for chains of water molecules are: 1,2,3/2, 5/3 ,8/5 and 13/8, respectively. These values are set of Fibonacci sequence of Φ. The question is what is the chain length of stable water molecules? The system of Fibonacci determinants provides the answer. The number of molecules where the determinant of non-covalent hydrogen bonds is equal to zero gives a stable chain. (Suppl. Figure 6). A chain of 3, 4, 5...8 water molecules would not be stable, it would be created and destroyed within a few *ps*. However, for a chain of nine water molecules (not beginning with a monomer or dimer water molecule, but with a trimer) the determinant of such a system is equal zero and chain is stable. Same case is with Fibonacci chain of 16 water molecules (Suppl. Figure 6).

Supplement Table 1 (ST1): Icosahedral symmetry group that determines the energy states (T_{1g}, T_{2g}, T_{1u} and T_{2u}) of structures and processes. Fibonacci numbers Φ, -Φ, ϕ, -ϕ are subset of icosahedral symmetry group (± ½(1+√5 and ± ½(1-√5)) (Adapt from Kettle, S.F.A., Symmetry and structure, John Willey and Sons, Chichester, 1995).

THE ICOSAHEDRAL GROUPS

Multiplication Table of the Groups I and I_h

I/I_h	E	$12C_5$	$12C_5^2$	$20C_3$	$15C_2$	i	$12S_{10}$	$12S_{10}^3$	$20S_6$	15σ	III	IV	
A_g	1	1	1	1	1	1	1	1	1	1		$x^2+y^2+z^2$	
T_{1g}	3	$\frac{1}{2}(1+\sqrt{5})$	$\frac{1}{2}(1-\sqrt{5})$	0	−1	3	$\frac{1}{2}(1-\sqrt{5})$	$\frac{1}{2}(1+\sqrt{5})$	0	−1	(R_x, R_y, R_z)		
T_{2g}	3	$\frac{1}{2}(1-\sqrt{5})$	$\frac{1}{2}(1+\sqrt{5})$	0	−1	3	$\frac{1}{2}(1+\sqrt{5})$	$\frac{1}{2}(1-\sqrt{5})$	0	−1			
G_g	4	−1	−1	1	0	4	−1	−1	1	0			
H_g	5	0	0	−1	1	5	0	0	−1	1		$(2z^2-x^2-y^2, x^2-y^2, xy, yz, zx)$	
A_u	1	1	1	1	1	−1	−1	−1	−1	−1			
T_{1u}	3	$\frac{1}{2}(1+\sqrt{5})$	$\frac{1}{2}(1-\sqrt{5})$	0	−1	−3	$-\frac{1}{2}(1-\sqrt{5})$	$-\frac{1}{2}(1+\sqrt{5})$	0	1	(x, y, z)		
T_{2u}	3	$\frac{1}{2}(1-\sqrt{5})$	$\frac{1}{2}(1+\sqrt{5})$	0	−1	−3	$-\frac{1}{2}(1+\sqrt{5})$	$-\frac{1}{2}(1-\sqrt{5})$	0	1			(x^3, y^3, z^3)
G_u	4	−1	−1	1	0	−4	1	1	−1	0			$[x(z^2-y^2), y(z^2-x^2), z(x^2-y^2), xyz]$
H_u	5	0	0	−1	1	−5	0	0	1	−1			

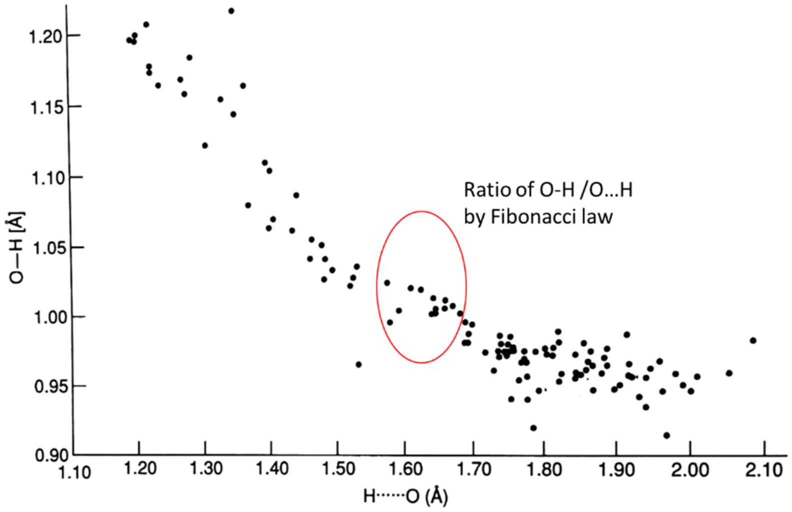

Supplement Figure 3 (S3): Plot of covalent (O-H) hydrogen bonds and non-covalent (O…H) hydrogen bonds based on neutron diffraction data (Jeffrey, G.A. and Saenger,W., Hydrogen Bonding in biological Structures, Springer-Verlag, Berlin, 1991.)

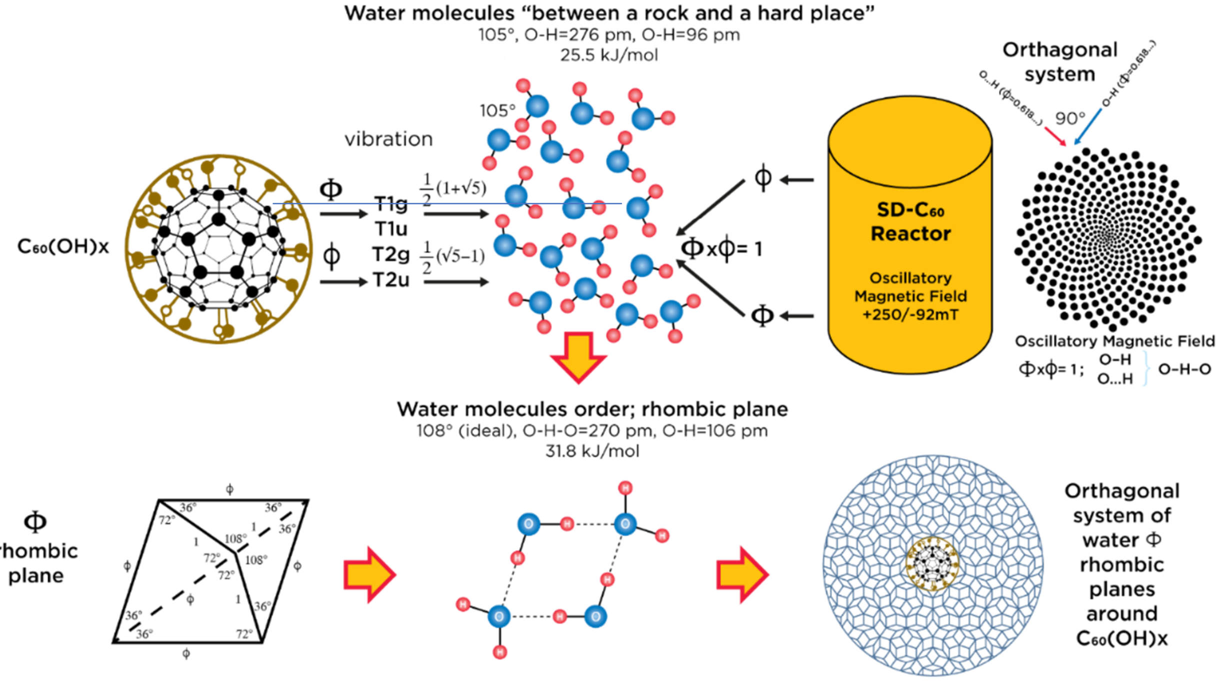

Supplement Figure 4 (S4): Schematic representation of the formation of an elastic soft-matter structure (SD-C_{60}), composed of water molecules that are elastic (deformable) and that form 3-dimensional icosahedral water shells (layers, onions) as a soft material.

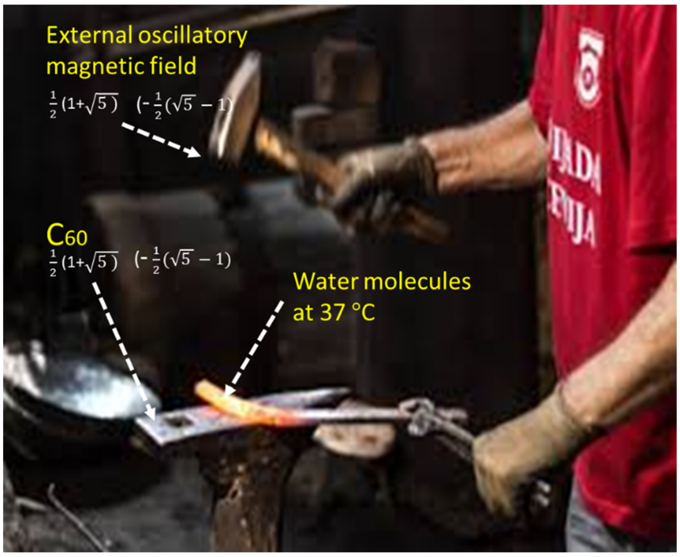

Supplement Figure 5(S5): The principle "between a hammer and an anvil" in process water tiles and shells (onion) formation according to action of C_{60} vibrations and external oscillatory magnetic field by icosahedron symmetry

$$Det(D3)_F = \begin{vmatrix} 3 & 5 & 8 \\ 13 & 21 & 34 \\ 55 & 89 & 144 \end{vmatrix} = 0 \qquad Det(D4)_F = \begin{vmatrix} 1 & 2 & 3 & 5 \\ 8 & 13 & 21 & 34 \\ 55 & 89 & 144 & 233 \\ 377 & 610 & 987 & 1597 \end{vmatrix} = 0$$

Supplement Figure 6 (S6): Two Fibonacci determinants *detF*(3) and *detF*(4) (set of $\{9,16\}_\Phi$) by which the oscillatory magnetic field is programmed to stimulate rhombic tiles formation . Since the determinant column is a linear combination of the remaining columns of that determinant, then the determinant is equal to zero (this follows from the law of the sequence: $u_{n-1} + u_n = u_{n+1}$) than the Φ-rhombic tiles of $\{9,16\}_\Phi$ set are stable.

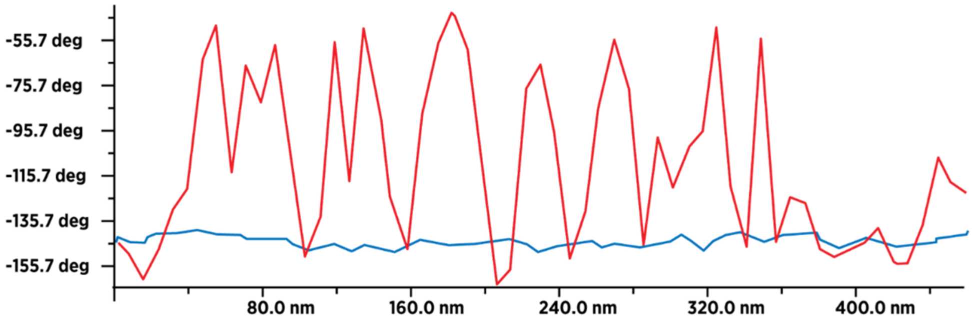

Supplement Figure 7 (S7): Presence water molecules in FD-C_{60} and SD-C_{60}. Comparative diagrams (from Figure 16 and 17, range 0-400 nm) of intensity values of dipole-dipole interactions between MFM magnetic tip and substrate (FD-C_{60} - blue line and SD-C_{60} - red line). FD-C_{60} has a low intensity of dipole-dipole interaction due to humidity, while SD-C60 has a high intensity of dipole-dipole interaction because, in addition to humidity, it contains water molecules packed in rhombic plates (3DPT).

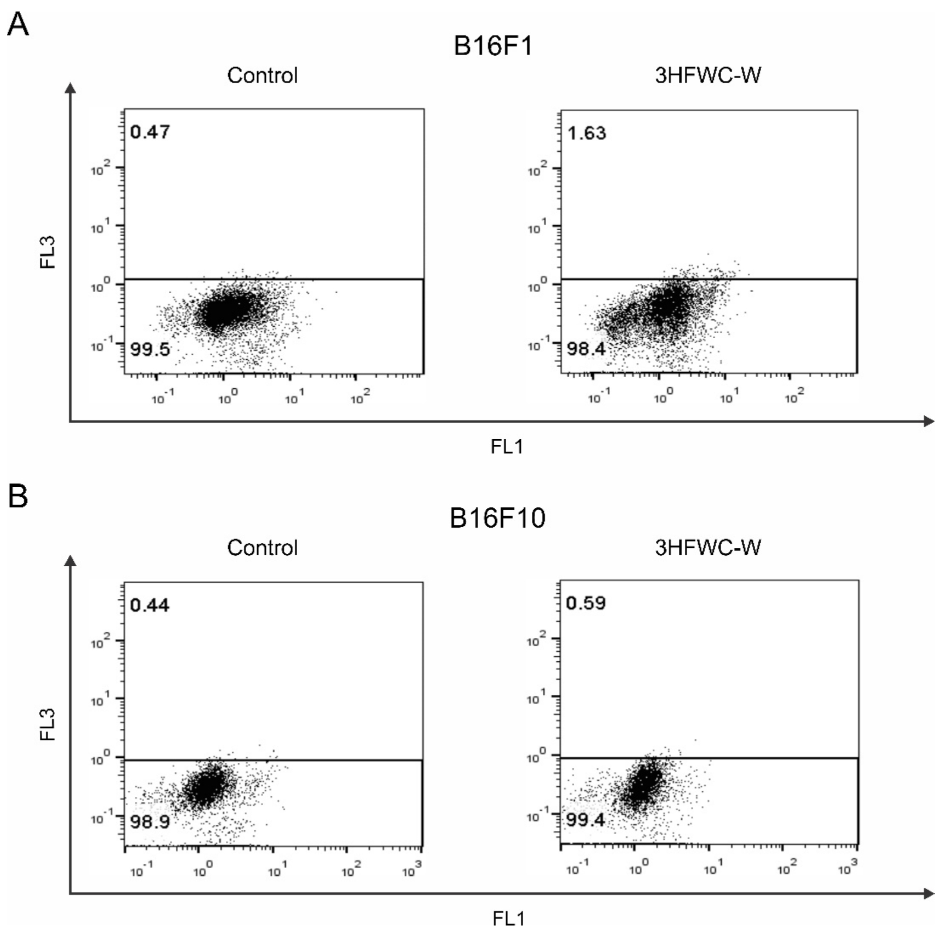

Supplement Figure 8 (S8). The effect of 3HFWC-W on induction of autophagy in melanoma cells. The percentage of cells in autophagy in B16F1 (A) and B16F10 (B) cell cultures treated with the IC_{50} value of 3HFWC-W (SD-C_{60}) for 24 h was determined by AO staining and subsequent flow cytometry. Representative dot plots of one out of three independently performed experiments are shown.

Supplement (**S9**): **Nanoparticle – nano gold:** Nanoparticles that are used today in biomedical research and their possible application in medicine can also have an influence on hydrogen bonds. However, a special attention for interaction with SD-C_{60} (3HFWC) has gold nanoparticles because of their plasmonic effects. Our initial research shows that several (12-18) spherical SD-C_{60} (size 10-15 nm) are packed around a gold nanoparticle (size 60-80 nm), enhancing the effects of collagen quality improvement, skin moisturization and the epidermis-dermis function (faster and better wound healing). Our explanation of this phenomenon is that there are better effects on collagen types I, III and VII in the basal membrane of the epithelial tissue. In order to give final conclusions about this synergistic effect of SD-C_{60}, gold nanoparticles and other nanoparticles, further research is necessary (Rudolf R, Jelen, Ž, Zadravec M, Majerič, P, Jović, Z, Vuksanović, M, Stankovic, I, Matija, L. Dragičević, A, Thompson NM, Horvat, A, Koruga D. A gold nanoparticles and hydroxylated fullerene water complex as a new product for cosmetics. *Advances in Production Engineering & Management.* 2022;17:89-107. https://doi.org/10.14743/apem2022.1.423)

Supplement: (S10) Symmetry bricking of hydrogen bonds functionality in DNA . The importance of hydrogen bonds for cell functionality (healthy/cancer) was discussed in a 2012 paper (Koruga, D., Classical and Quantum Information Processing in DNA-Protein Coding, in book , Cell and Tissue

Engineering, Ed. Obradovic, B., Springer, 2012, ISBN: 978-3-642-21912-2) It has been shown that the impairment of functionality primarily depends on the symmetry of hydrogen bonds in DNA, which can be impaired under the influence of various external factors. One of the factors is inadequate signal transmission (via water) to the phosphor group of DNA. Two basic states "-1L" and "+1R" can occur (as shown in the picture).

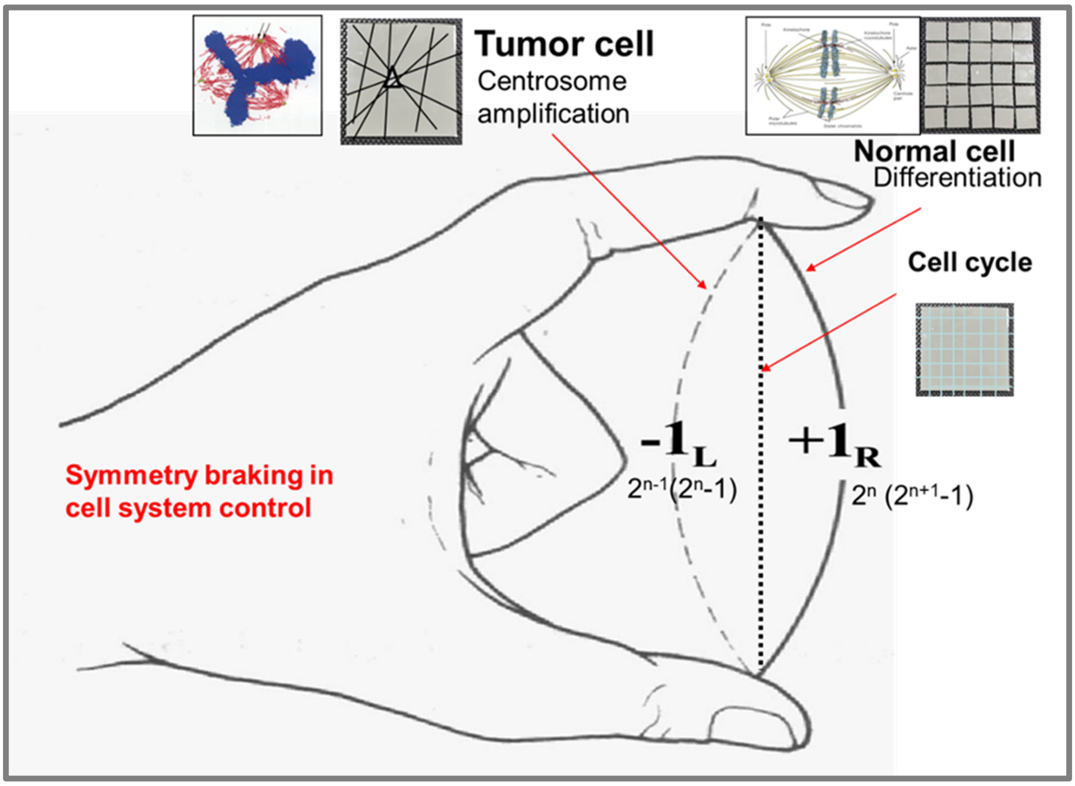

Figure 9 (S10): Symmetry bricking of hydrogen bonds functionality in DNA. During cell cycle the symmetry bricking of hydrogen bonds in cell system control. During the cell cycle, breaking the symmetry of the functionality of the hydrogen bonds in DNA leads to dysfunctionality, which leads to cell death or cancer. Returning the cell to its normal state is possible if reprogramming is performed (returning to a harmonized state of functioning of hydrogen bonds). This mechanism is realized if the resonance relationship of the hydrogen bonds is harmonized, i.e. if the external vibrations of the hydrogen bonds (via PO4) fit with the vibrations in DNA (A=T, C=G). Koruga, D., Classical and Quantum Information Processing in DNA-Protein Coding, in book , Cell and Tissue Engineering, Ed. Obradovic,B.,Springer, 2012, https://www.researchgate.net/publication/233796030).
